# Supplementary material for: Morphological and molecular barcode analysis of the medicinal tree Mimusops coriacea (A.DC.) Miq. collected in Ecuador
Source: PeerJ. 2019 Oct 11;7:e7789. doi: 10.7717/peerj.7789 (PMC6791349; doi:10.7717/peerj.7789)
Supplement: Supplemental Information 1 [file peerj-07-7789-s001.pdf]

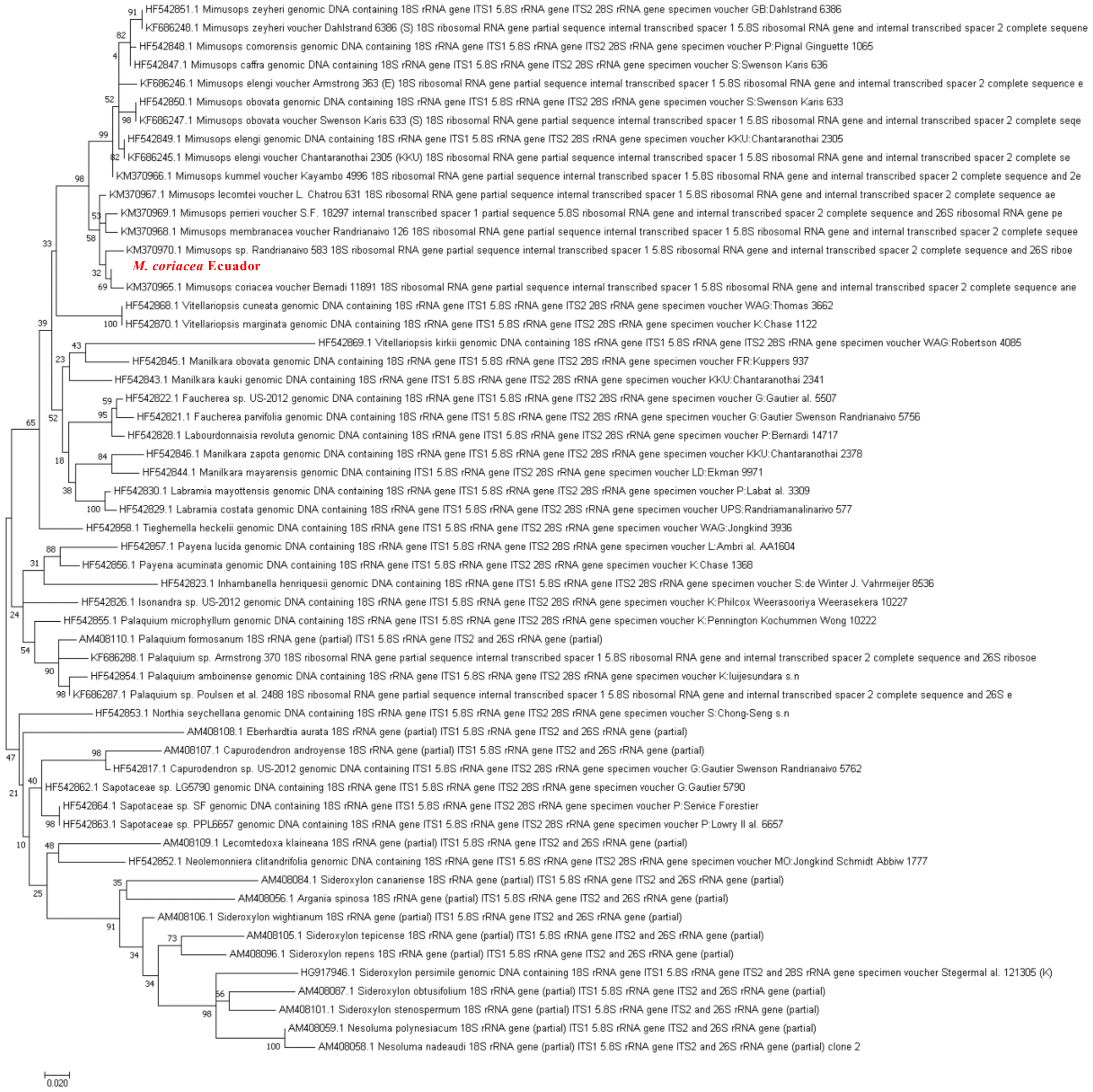

Phylogenetic tree of the ITS1 with accessions from the Subfamily Sapotoideae. Maximum likelihood method based Tamura 3- parameter. Bootstraap test with 100 replicates was performed. A discrete Gamma distribution was used to model evolutionary rate differences among sites (5 categories (+*G*, parameter = 0.8628)). The tree is drawn to scale, with branch lengths measured in the number of substitutions per site. The analysis involved 57 nucleotide sequences. All positions containing gaps and missing data were eliminated. There were a total of 235 positions in the final dataset. Evolutionary analyses were conducted in MEGA7.

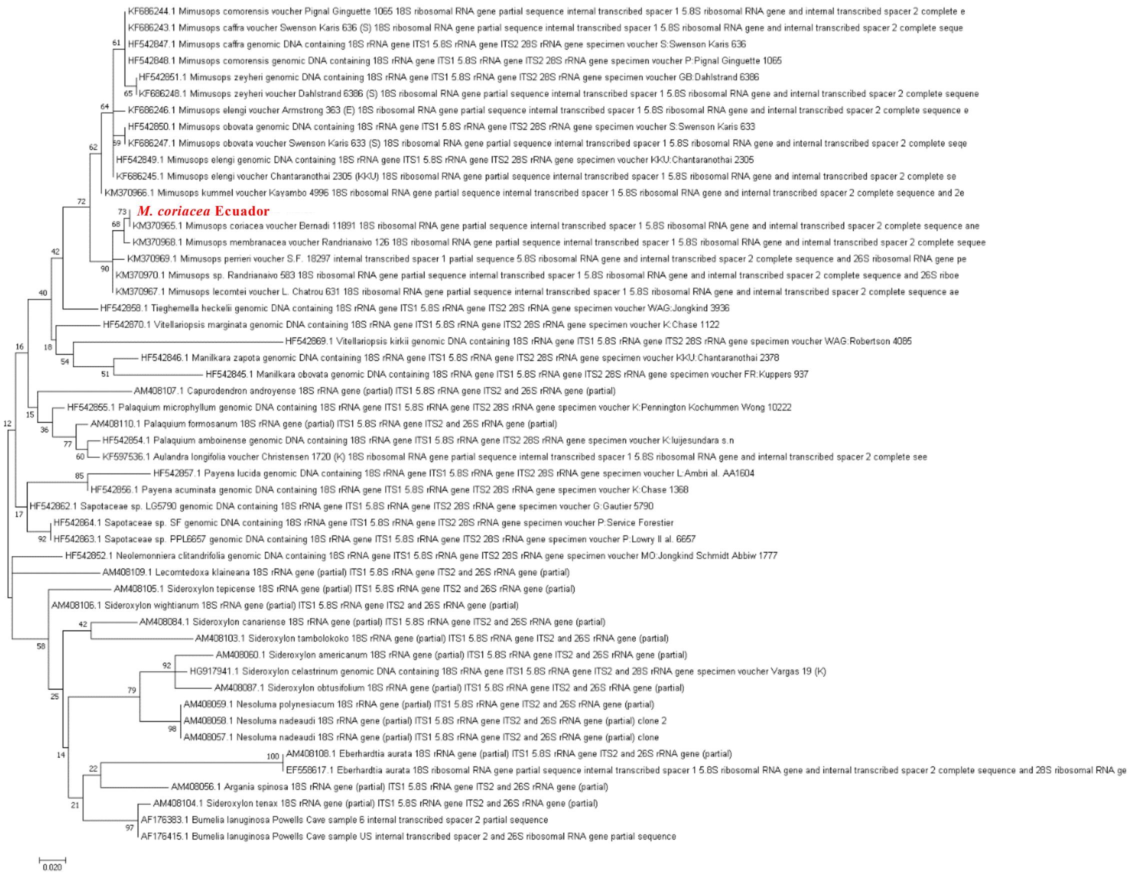

Phylogenetic tree of the ITS2 with accessions from the Subfamily Sapotoideae. Maximum likelihood method based Tamura 3- parameter. Bootstrap test with 100 replicates was performed. A discrete Gamma distribution was used to model evolutionary rate differences among sites (5 categories (+*G*, parameter = 0.9476)). The tree is drawn to scale, with branch lengths measured in the number of substitutions per site. The analysis involved 51 nucleotide sequences. All positions containing gaps and missing data were eliminated. There were a total of 108 positions in the final dataset. Evolutionary analyses were conducted in MEGA7.

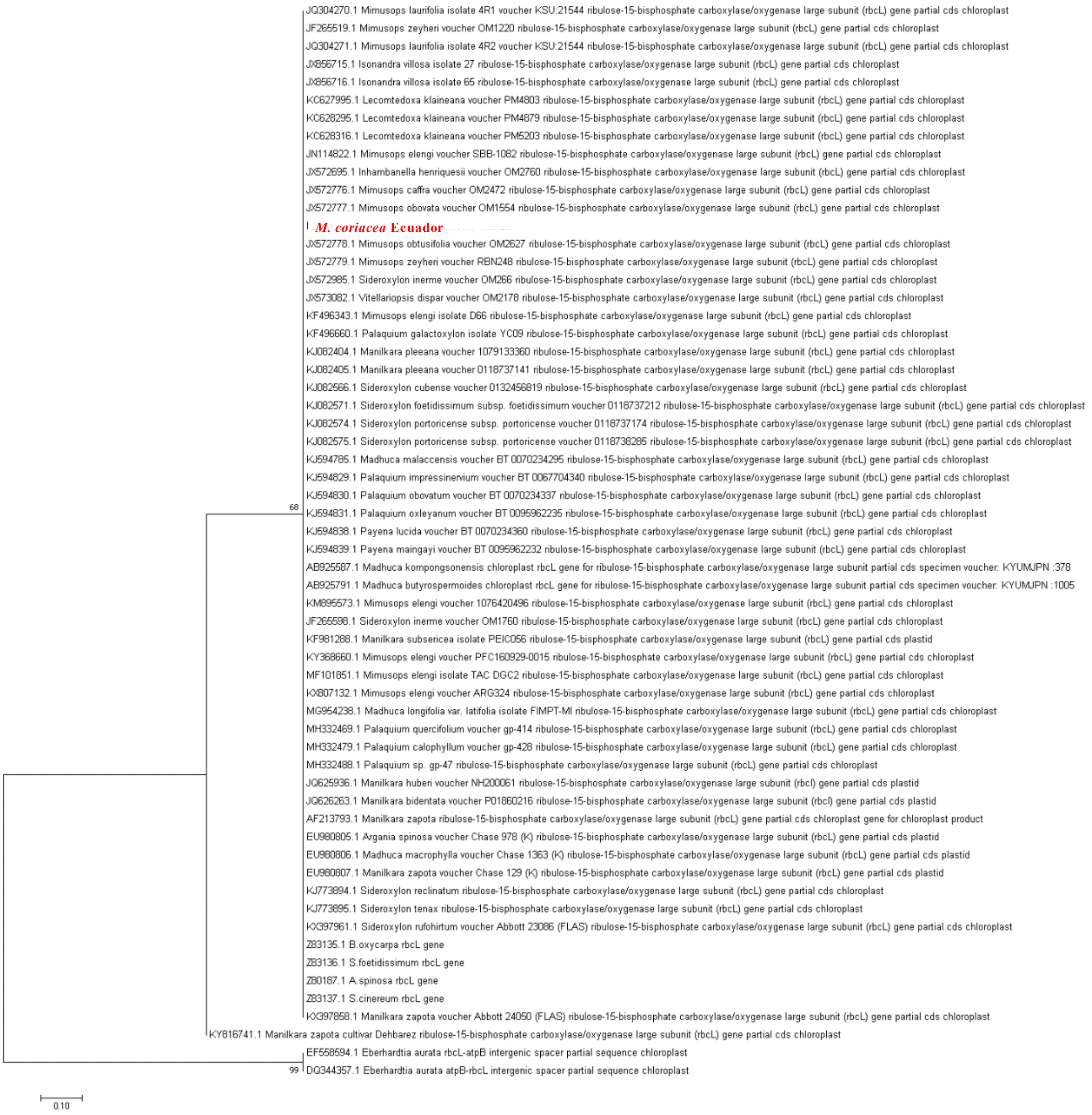

Phylogenetic tree of the *rbc*L with accessions from the Subfamily Sapotoideae. Maximum likelihood method based Jukes-Cantor model. Bootstrap test with 100 replicates was performed. The tree with the highest log likelihood (-17.19) is shown. The percentage of trees in which the associated taxa clustered together is shown next to the branches. Initial tree(s) for the heuristic search were obtained automatically by applying Neighbor-Join and BioNJ algorithms to a matrix of pairwise distances estimated using the Maximum Composite Likelihood (MCL) approach, and then selecting the topology with superior log likelihood value. The tree is drawn to scale, with branch lengths measured in the number of substitutions per site. The analysis involved 60 nucleotide sequences. Codon positions included were 1st+2nd+3rd+Noncoding. All positions containing gaps and missing data were eliminated. There were a total of 5 positions in the final dataset. Evolutionary analyses were conducted in MEGA7.

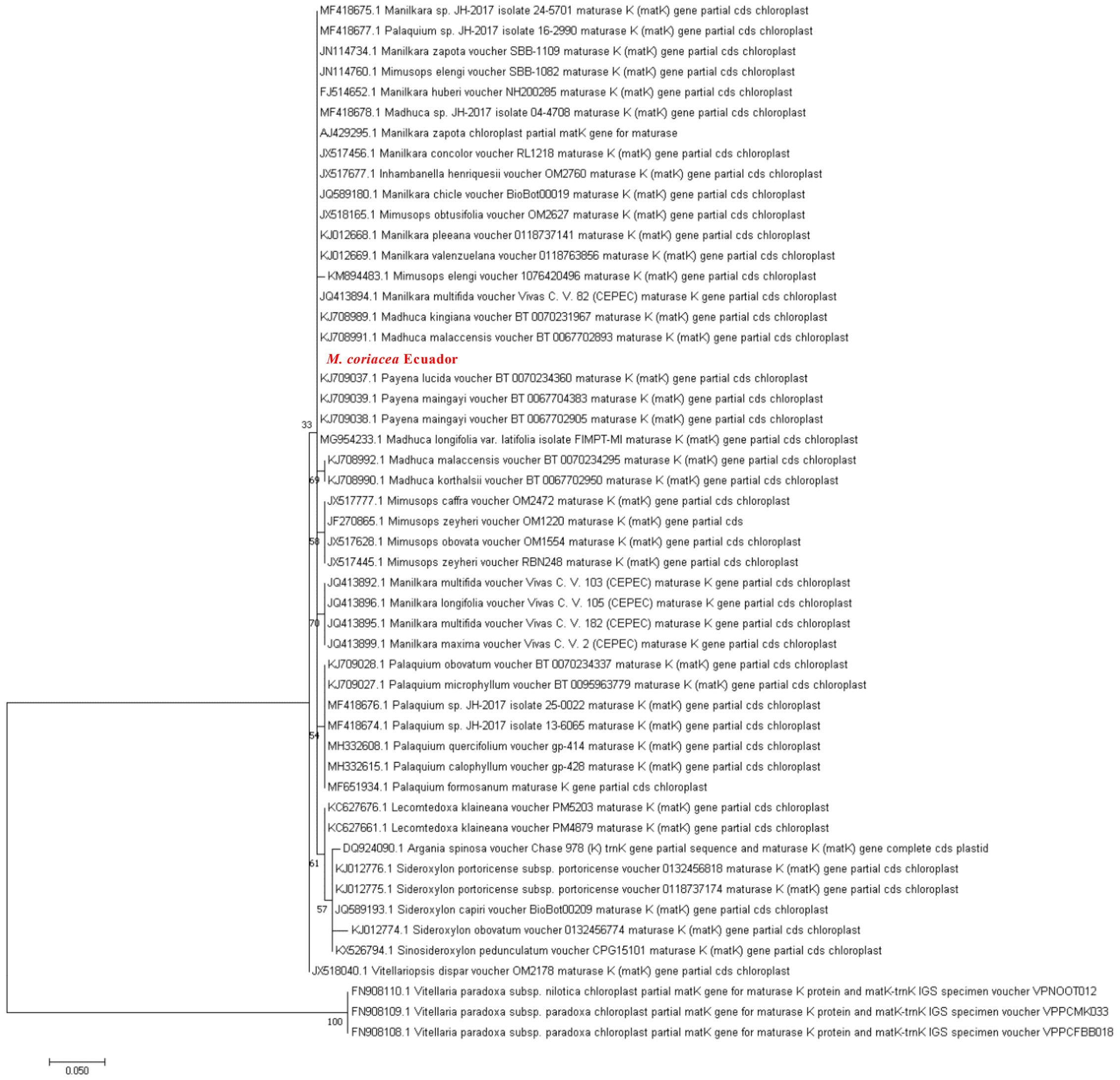

Phylogenetic tree of the *mat*K with accessions from the Subfamily Sapotoideae. Maximum likelihood method based Tamura 3- parameter. Bootstrap test with 100 replicates was performed. The tree with the highest log likelihood (-418.50) is shown. The percentage of trees in which the associated taxa clustered together is shown next to the branches. Initial tree(s) for the heuristic search were obtained automatically by applying Neighbor-Join and BioNJ algorithms to a matrix of pairwise distances estimated using the Maximum Composite Likelihood (MCL) approach, and then selecting the topology with superior log likelihood value. The tree is drawn to scale, with branch lengths measured in the number of substitutions per site. The analysis involved 51 nucleotide sequences. Codon positions included were 1st+2nd+3rd+Noncoding. All positions containing gaps and missing data were eliminated. There were a total of 144 positions in the final dataset. Evolutionary analyses were conducted in MEGA7.
