# Supplementary material for: Morphological and molecular barcode analysis of the medicinal tree Mimusops coriacea (A.DC.) Miq. collected in Ecuador
Source: PeerJ. 2019 Oct 11;7:e7789. doi: 10.7717/peerj.7789 (PMC6791349; doi:10.7717/peerj.7789)
Supplement: Supplemental Information 2 [file peerj-07-7789-s002.pdf]

Data S1

> M.coriacea_RBCL
GGTACATGGACAACTGTGTGGACCGATGGACTNACTAGCCTTGATCGTTACAAAGGGCGATGCTACCAC
ATCGAGCCCGTTGCTGGAGAAGAAACTCAATTTATTGCTTATGTAGCTTATCCTTTAGACCTTTTTGAAGA
AGGTTCTGTTACTAATATGTTTACTTCCATTGTGGGGAATGTATTTGGGTTCAAAGCCCTGCGCGCTTTAC
GTCTGGAAGATCTGCGAATCCCTCCTGCGTATTCTAAAACTTTCCAAGGACCGCCTCATGGCATCCAAGT
TGAAAGAGATAAATTGAACAAGTACGGTCGTCCCCTGTTGGGATGTACTATTAAACCTAAATTGGGGTT
ATCTGCTAAAAACTACGGTAGAGCGGTTTATGAATGTCTCCGCGT

> M.coriacea _MATK
TTTTCTCCGCAACCAATCTTCTCATTTACGATCAATATCTTCTGGAACCTTTTTTGAACAAATAGATTTCTA
TGAAAAAATAGAACATTTTGTAGAAGTCTTTACTAAGGATTTTCAGGCCATTCTATGGTTGTGCAAGGAT
CCTTTCATGCATTATATTAGGTATCAAGGAAAATCCCTTTTGGCATCAAAAGGGAAGTCCCTTTTGATGA
ATAAATGGAAATATTACTTTGTCAATTTCTGGCAATGTTATTTTTACATGTGGTCTCAACCAGGAAGAATC
CATATAAACCAATTATCCAATCATTCCTTCGACTTTCTGGGCTATCTTTCAAGTGTGCGACTAACCCCTTCA
ATGGTACGGAGTCAAATGCTAGAAAATTCATTTCTAATAGGTAATGCTATTAAGAAGTTCGATACCCTAG
TTCCAATTATTCCTCTGATTGGATCATTGTCTAAAGCGAAATTTTGTAACGTATTAGGGCATCCCATTAGT
AAGCCAGTCTGGGCTGATTTATCAGATTCTGATATTATTGACCGATTCGGGCGTATATATAGAAATCTTT
CTCATTATTATAGCGGGTCCTCAAAAAAAATGAATTTGTA

>M.coriacea_ITS_I
GCGGAAGGATCATTGTCAAAAACCTGCCAAGCAGAACAACCCGCGAACTTGTATAGTAACCACTGGGG
GGTCCTTGTGCCCCTCGCCAGGTGCGCTCTTGAATTGCGTTGCCTCGGCTAAAAACTTAACCCCGACGCG
AATTCGCGTCAAGGAACTTTAACAAGAAGAGCGCCCCCTGCTCTCGTTCGCGAGTGTGAAGTTACGGGG
GCTAGTCGCATCTTTTTATGAACATAACGACTCTCGGCAACGGATATCTCGGCTCTCGCATCGATGAAGA
ACGTAGCGAAATGCGATACTTGGTGTGAATTGCAGAATCCCGTGAACCATCGAGTCTTTGAACGCAAGT
TGCGCCCGAAGTCATTAGGCCGAGGGCACGCCTGCCTGGGCGTCTCGCATCGCGTCGCCCCCCACCTTT
GCCCGCGGATTTTGGGTTGGTGGGGGCGGAATTTGGCCCCCCGTGTGCTGTAGTGCGCGGTTGGCCTA
AATTTAAGTCCCGGGCGTCAAACGTCACGACGAGTGGTGGATGTGATACTCTTGCGTCATGTCGTGCAC
GTCTTTATGGACCCCCAGGATTGTTTCGTTTTTGACCCTGAAGCACCGTTTTCCACGGAGCACTCGATCGC
GACCCCAGGTCAGG

> M.coriacea_ITS_2
GAATCCCGTGAACCATCGAGTCTTTGAACGCAAGTTGCGCCCGAAGTCATTAGGCCGAGGGCACGCCTG
CCTGGGCGTCTCGCATCGCGTCGCCCCCCACCTTTGCCCGCGGATTTTGGGTTGGTGGGGGCGGAATTT
GGCCCCCCGTGTGCTGTAGTGCGCGGTTGGCCTAAATTTAAGTCCCGGGCGTCAAACGTCACGACGAGT
GGTGGATGTGATACTCTTGCGTCATGTCGTGCACGTCTTTATGGACCCCCAGGATTGTTTCGTTTTTGACC
CTGAAGCACCGTTTTCCACGGAGCACTCGATCGCGACCCCAGGTCAGGCGGGATTACCCGCTGAGTTTA

AGCATATCAATAAGCGGAGGAAAAGAAACTTACAAGGATTCCCTTAGTAACGGCGAGCGAACCGGGAA
TAGCCCAGCTGAAAAATCGTGCGGCGTCGCCGTTCGAATTGTAGTCTG
